# Redox signaling and unfolded protein response coordinate cell fate decisions under ER stress

**DOI:** 10.1016/j.redox.2018.11.005

**Published:** 2018-11-14

**Authors:** Zhe Zhang, Lu Zhang, Li Zhou, Yunlong Lei, Yuanyuan Zhang, Canhua Huang

**Affiliations:** a Department of Biotherapy, State Key Laboratory of Biotherapy and Cancer Center, West China Hospital, and West China School of Basic Medical Sciences & Forensic Medicine, Sichuan University, and Collaborative Innovation Center for Biotherapy, Chengdu 610041, PR China; b Department of Pharmacology, West China School of Basic Medical Sciences & Forensic Medicine, Sichuan University, Chengdu 610041, PR China; c Department of Biochemistry and Molecular Biology, and Molecular Medicine and Cancer Research Center, Chongqing Medical University, Chongqing 400016, PR China

**Keywords:** APX, Ascorbate peroxidase, ATF4, Activating Transcription Factor 4, ATF6α, activating transcription factor 6α, ASK1, apoptosis signal-regulating kinase 1, CHOP, CAAT/enhancer binding protein (C/EBP) homologous protein, CRAC, Ca^2+^ release-activated Ca^2+^, ER, endoplasmic reticulum, eIF2α, eukaryotic translation initiation factor 2α, ERAD, ER-associated degradation, ERO1, ER oxidoreductin-1, GADD34, DNA damage gene 34, GPx7/8, glutathione peroxidase 7/8, GSH, reduced glutathione, GSSG, oxidized glutathione, GSTP, Glutathione S-Transferase P, IP3, inositol-1,4,5-trisphosphate, IP3R, inositol-1,4,5-trisphosphate receptor, IRE1α, inositol-requiring protein 1α, JNK, c-Jun NH2-terminal kinase, MAM, mitochondrial-associated membranes, NOX, NADPH oxidase, NRF2, nuclear factor erythroid 2-related factor 2, OPF, Oxidative protein folding, p-AKT, phosphorylated protein kinase B, PDI, protein disulfide isomerase, p-ERK, phosphorylated extracellular signal-regulated kinase, PERK, protein kinase RNA-like ER kinase, PRDX4, peroxiredoxin 4, QSOX, quiescin sulfhydryl oxidase, RIDD, regulated IRE1-dependent decay, ROS, reactive oxygen species, S1P, site-1 protease, S2P, site-2 protease, SERCA, sarcoplasmic/endoplasmic reticulum Ca^2+^-ATPases, SOCs, store operated Ca^2+^ channels, STIM1, stromal interaction molecule 1, TRAF2, TNF receptor-associated factor 2, TXNIP, thioredoxin interacting protein, UPR, unfolded protein response, VKOR, Vitamin K epoxide reductase, Vps34, vacuolar protein sorting 34, XBP1, X-box binding protein 1, Redox regulation, ER stress, UPR, Cell fate

## Abstract

Endoplasmic reticulum (ER) is a dynamic organelle orchestrating the folding and post-translational maturation of almost all membrane proteins and most secreted proteins. These proteins synthesized in the ER, need to form disulfide bridge to acquire specific three-dimensional structures for function. The formation of disulfide bridge is mediated via protein disulfide isomerase (PDI) family and other oxidoreductases, which contribute to reactive oxygen species (ROS) generation and consumption in the ER. Therefore, redox regulation of ER is delicate and sensitive to perturbation. Deregulation in ER homeostasis, usually called ER stress, can provoke unfolded protein response (UPR) pathways with an aim to initially restore homeostasis by activating genes involved in protein folding and antioxidative machinery. Over time, however, activated UPR involves a variety of cellular signaling pathways which determine the state and fate of cell in large part (like autophagy, apoptosis, ferroptosis, inflammation, senescence, stemness, and cell cycle, etc.). This review will describe the regulation of UPR from the redox perspective in controlling the cell survival or death, emphasizing the redox modifications of UPR sensors/transducers in the ER.

## 1 Introduction

The endoplasmic reticulum (ER) is the widest intracellular organelle spanning from the nuclear envelope to the cell membrane. It is mainly responsible for the correct folding and posttranslational modification of proteins destined to other organelles, the plasma membrane, as well as the extracellular compartment. In addition, it is also deputed to several different activities, including calcium storage, detoxification of chemical compounds, and lipid synthesis. Oxidative protein folding (OPF) characterized by intermolecular or intramolecular disulfide bond formation, the most common post-translational modification which performs in the ER, is a major source of H_2_O_2_ of the ER [1], [2], [3]. For instance, ER oxidoreductin-1 (ERO1) proteins, cooperating with PDIs which play important roles in OPF, provides an answer to how oxidative power can be generated in the ER [4], [5]. Same as ERO1, quiescin sulfhydryl oxidase (QSOX) is involved in the OPF to generate H_2_O_2_ [6]. Including OPF, reactive oxygen species (ROS) may be from the catalytic process in charge of NADPH oxidases 4 (NOX4) [7], or respiratory chain of mitochondrion [8], [9]. To maintain the redox homeostasis of the ER, H_2_O_2_, as an common oxidant, is consumed by some oxidoreductases (such as peroxiredoxin 4 (PRDX4), glutathione peroxidase 7/8 (GPx7/8), and ascorbate peroxidase (APX)) to ensure the OPF in order [10], [11], [12]. In addition, reduced glutathione (GSH), the most common reducing granules in cells, also contributes to the removal of excessive ROS [13], [14], [15]. The homeostasis of ROS in the ER is vital. Although the oxidative environment is conducive to OPF, excessive accumulation of ROS (named oxidative stress) can destroy the redox homeostasis of the ER, leading to accumulation of the misfolded proteins that cause ER stress [3], [16]. ER stress and oxidative stress usually interact and crosstalk with each other.

When perturbation occurs in ER homeostasis (protein folding homeostasis or redox homeostasis), the unfolded protein response (UPR) is activated to restore stress [17], [18], [19]. UPR sensors, including inositol-requiring protein 1α (IRE1α), protein kinase RNA-like endoplasmic reticulum (ER) kinase (PERK) and activating transcription factor 6α (ATF6α), transduce information of ER status to the cytosol and nucleus to restore protein-folding capacity by transcribing a series of related-genes, and are the redox sensors revealed by constant research [20]. UPR is able to induce a complex signaling pathway networks, including either pro-survival mechanisms involving autophagy, antioxidant response, ER-associated degradation (ERAD) and ER biogenesis, etc. [21], or pro-death mechanisms involving apoptosis and ferroptosis, etc. [22]. Growing evidence has emerged showing that ROS and redox signaling are deeply involved in the determination of cell state and fate.

This review will illustrate how the ER maintains redox homeostasis, discuss in detail how related sensors are redox modified to participate in UPR during ER stress, and summarize the relationship between ER stress and redox regulation in cellular processes to clarify how redox regulation in the ER determines the fate of cells.

## 2 Protein folding homeostasis and redox homeostasis in the ER

**Fig. 1:**
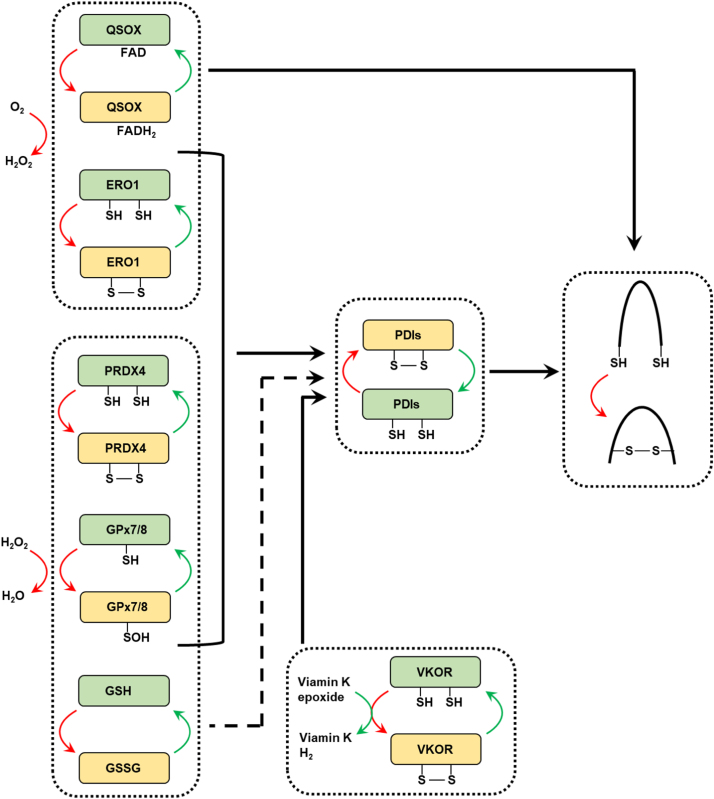
**Protein disulfide bond formation in the ER.** Formation of disulfide bond in substrate proteins is mainly mediated by PDI family members. PDIs are oxidized to form a disulfide bond, then the disulfide bond is introduced to substrates, and PDIs are simultaneously reduced. ERO1, PRDX4, GPX7/8 and VKOR contribute to the re-oxidization and reactivation of PDIs. ERO1 is able to catalyze oxidation reaction by coupling *de novo* disulfide formation to the reduction of oxygen to H_2_O_2_. PRDX4 and GPX7/8 utilize H_2_O_2_ as oxidant, then oxidized PRDX4 and GPX7/8 is able to reactive PDIs. VKOR can accept electrons from PDIs to regenerate vitamin K. QSOX uses oxygen to contribute to disulfide formation of substrate proteins directly and H_2_O_2_ production from oxygen. The oxidized glutathione (GSSG) also plays an important role in maintaining ER redox homeostasis. Red arrows indicate the flow of oxidizing equivalents, while green arrows indicate the flow of reducing equivalents. Dashed-lined arrows indicate the potential function in biochemistry, but the function of these molecular still lack solid cell biological evidence in mammalian cells so far.

**Fig. 2:**
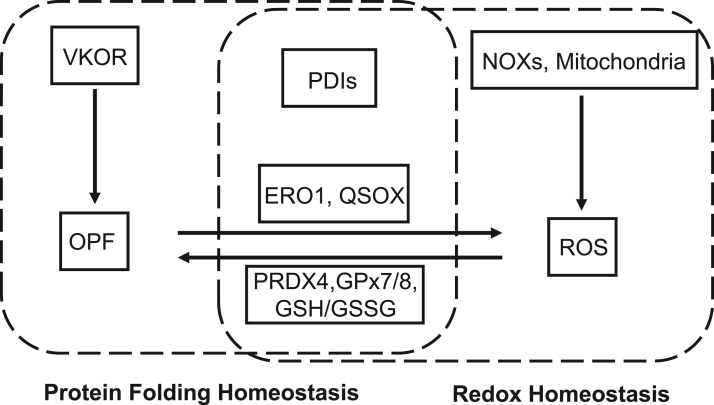
**Crosstalk between redox homeostasis and protein folding homeostasis in the ER.** OPF is deeply associated with redox balance in the ER. ROS (mainly H_2_O_2_) can be generated during OPF process through ERO1 and QSOX, and from NOXs in the ER membrane, as well as ER-associated mitochondria. ER-resident PRDX4, GPx7/8 are oxidized by H_2_O_2_ and further oxidize PDIs, simultaneously consume H_2_O_2_. VKOR facilitate OPF without affecting ROS.

A common feature of proteins is the requirement for a specific three-dimensional structure to implement the biological function [1], [2]. The native conformation is stabilized by intramolecular disulfide bonds for many proteins, especially for secretory and membrane proteins [23]. The ER is a reticular organelle where nascent proteins translocated from cytoplasmic ribosome are folded and modified to be functional (Fig. 1) [24]. During protein folding process, H_2_O_2_ is produced as a byproduct in the ER, which maintains the relatively high levels of ROS [4], [5]. Due to the disulfide bridge formation in the protein folding process, ER redox state is closely linked to ER protein homeostasis and proper functioning of the ER (Fig. 2).

### 2.1 OPF and H_2_O_2_ generation

OPF is characterized by intramolecular disulfide bond formation, an oxidative process and probably the most common post-translational modification [25]. The disulfide bond formation is primarily catalyzed by PDI [26], [27], which possesses four Trx-like domains (a, a′, b, b′) and a c-domain with a KDEL ER retention sequence [28], [29]. In PDI, the redox state of CGHC motifs in the a-domains dictate if oxidase or isomerase chemistry is performed [30], [31]. The non-catalytic b′ domain is responsible for identifying unfolded and improperly folded proteins through exposed hydrophobic patches on the nascent protein [32]. For the catalytic function, oxidation of nascent proteins is accomplished via the reduction of PDI mediated by CGHC active-site [31]. Subsequent re-oxidation of the PDI active sites carries out the next reaction [33]. The primary source of oxidative power is O_2_, which is utilized by ER oxidoreductases to oxidize PDI and generate H_2_O_2_ as a byproduct. H_2_O_2_ can be used to form disulfide bond in peroxidases, which is able to re-oxidize PDI, or scavenged by ER antioxidant system to maintain redox homeostasis in the ER (Fig. 1).

#### 2.1.1 ER oxidoreductin-1

Studies have reported that the net oxidative power required for disulfide bond formation comes from O_2_ by ERO1 [34], [35]. ERO1 is a highly conserved flavoprotein containing two cysteine pairs, one locates on a flexible loop and the other is as part of a CXXC motif proximal to the FAD cofactor [34]. It uses O_2_ as acceptor of electrons from sulfydryls, catalyzes PDI to form disulfide bond, meanwhile leading to H_2_O_2_ generation. This process is very important to oxidize reduced PDI to insure the isomerase activity of PDI [4], [5]. Yeast Ero1p and the mammalian homologues ERO1α and ERO1β, are all tightly regulated to prevent overproduction of ROS to maintain the redox or protein folding homeostasis of ER (Fig. 1) [28], [36], [37], [38].

#### 2.1.2 Quiescin sulfhydryl oxidases

While ERO1 is essential in yeast to oxidize protein dithiols, double knockout of ERO1α and ERO1β only exhibits mild phenotype of ERO1β deficiency which compromises oxidative folding of proinsulin in mammals [39], [40]. This unexpectedly observation raises the question of what sustains oxidative folding in the absence of these flavoproteins. QSOX is a flavoprotein that contain an Erv domain fused with a Trx-like domain [6]. Similar to ERO1, QSOX catalyzes disulfide formation by coupling disulfide oxidation to the reduction of oxygen to form H_2_O_2_ (Fig. 1). However, unlike ERO1, which only oxidizes PDIs, QSOX is able to introduce disulfide bond into a broad spectrum of protein substrates [28], [36], [37], [38]. Additionally, due to its special structure, it may be able to bypass the disulfide exchange reaction catalyzed by PDI [6], [41]. In vitro, QSOX can shuttle disulfides between the Erv domain and the first thioredoxin domain, which can then exchange its disulfide with substrate proteins [42]. Overexpression of human QSOX in yeast can rescue the loss of viability and oxidative folding defects resulted from impaired Ero1p function [43]. These studies suggest that QSOX contribute largely to the oxidative protein folding within the ER in mammals.

### 2.2 Other sources of ROS in the ER

Mitochondria is the main source of ROS, which are generated from the electron transport chain. It has been reported that ER and mitochondria can contact closely through the mitochondrial-associated membranes (MAM) (Fig. 2) [8], [9]. ROS generated in the mitochondria can diffuse from mitochondria to the ER, where they participate in the redox homeostasis. NOXs are an important source of ROS [44]. Several NOXs locate at the ER membrane, where they catalyze the generation of ROS [44]. For instance, NOX4 has been shown to locate at the ER membrane and produce H_2_O_2_, which may be involved in activating apoptosis after prolonged ER stress (Fig. 2) [7].

### 2.3 OPF and ROS consumption

The ER H_2_O_2_ generated by ERO1 and/or QSOX during OPF, together with other H_2_O_2_-generating reactions (discussed below), can be utilized as an oxidizing power for OPF. This oxidizing reaction is mainly mediated by several peroxidases. Of note, ER glutathione which acts as a ROS scavenger, may be involved in OPF.

#### 2.3.1 Peroxidases

The mammalian ER is endowed with three known peroxidases: PRDX4 and two homologous GPx7/8 [10], [12], [45], [46]. PRDX4 is an ER-resident peroxiredoxin. Structurally, PRDX4 is a pentamer of dimers whose catalytic mechanism is based on a conserved peroxidative cysteine (C_P_) and a resolving cysteine (C_R_) [11], [47], [48]. In the catalytic cycle between PRDX4 and PDIs, C_P_ transfers two electrons to H_2_O_2_ to form cysteine sulfenic acid (C_P_-SOH). This sulfenylated cysteine can then react with a C_R_ present on an adjacent polypeptide to form a disulfide. There is one peroxidatic and one resolving cysteine per subunit, therefore two disulfides can form per dimer. Finally, this dimer is reduced by reduced PDIs in order to reestablish peroxidase activity (Fig. 1) [36], [49], [50]. However, disulfide bonded PRDX4 is efficiently reduced by some, but not all PDI family members. Another two peroxidases, GPx7/8 combined with PDI are efficient for peroxide-mediated OPF (Fig. 1). There is a physical association between ERO1 and both GPx7/8, and GPx7 is shown to increase the rate of oxygen consumption by ERO1α (as a measure of PDI oxidation) in vitro [51]. These observations indicate that PRDX4 and GPx7/8 constitute a substantial system by coupling OPF to ROS consumption.

#### 2.3.2 Glutathione (GSH/GSSG)

The ratio of GSH to GSSG in the cytoplasm is > 50:1, whereas that in the ER is < 3:1 [13]. Depletion of GSH by BSO treatment results in accelerated formation of disulfide bonds, but the disulfide bonds are non-native [52]. It has also been demonstrated that GSH competes with folding substrates for oxidation by ERO1 [53], which may contribute to the high ratio of GSSG to GSH in the ER. These observations suggest an important role of glutathione as a reducing power (Fig. 1). Notably, the glutathione redox state (GSH/GSSG ratio) plays an important role in PDI1-mediated ERO1 activation (reduction) and inactivation (oxidation) in yeast, which prevents excessive ERO1 activity and PDI oxidation [54], [55], [56], therefore maintaining ER folding homeostasis. Interestingly, fluorescent thiol-reacting agent-based assay revealed that in the ER, over half of the glutathione exists as oxidized glutathione or mixed disulfides with proteins, implying a more oxidative state than previous thought [57]. Despite these observations suggesting the important role of glutathione in the ER, cytosolic thioredoxin system has recently been shown to be critically required for proper ER OPF in mammals [58]. The function glutathione in the ER OPF has been elegantly reviewed in Ref. [59].

Besides the function of preventing an excessively oxidative environment in the ER, glutathione is important for post-translational modulation of ER proteins via S-glutathionylation. The glutathionylation in the ER is mediated by Glutathione S-Transferase P (GSTP), which is vital for the maintaining of ER homeostasis and UPR through glutathionylating multiple ER-resident proteins, including BiP, PDI, calnexin, calreticulin, endoplasmin, and sarco/endoplasmic reticulum Ca^2+^-ATPase (SERCA) [60]. Loss of GSTP enhances the sensitivity to ER stress, indicating that glutathione pool and S-glutathionylation of ER proteins are tightly associated with the ER function. These studies indicate that the ER glutathione is vital for redox homeostasis as well as protein folding via constituting a buffering milieu and S-glutathionylation modification.

### 2.4 OPF unrelated to ROS

#### 2.4.1 Vitamin K epoxide reductase

Vitamin K epoxide reductase (VKOR) is an ER membrane protein which can reduce vitamin K to support the carboxylation and consequent activation of vitamin K-dependent proteins [61], [62]. The activity of VKOR to reduce vitamin K epoxide involves Cys132 and Cys135, which is oxidized with concomitant vitamin K reduction and VKOR inactivation [63]. VKOR contains another two conserved cysteines (Cys43 and Cys51), which are not dispensable for the activity of vitamin K epoxide reduction. Instead, a disulfide bond between Cys43 and Cys51 is reduced by a redox protein to generate free sulfydryls, which in turn reduce the Cys132–Cys135 disulfide bond to reactivate VKOR [64]. Interestingly, VKOR is proposed to be able to accept electrons from PDI (Fig. 1) [65]. Recently, several PDI family members, TMX, TMX4, and ERp18 are uncovered to form an intermolecular disulfide bond with VKOR in bacteria [66]. These studies strongly suggest that VKOR can re-oxidize PDIs for OPF. However, solid evidence for the disulfide formation in PDIs remains insufficient, and the source of the original oxidizing power to oxidize Cys43–Cys51 in VKOR remains unknown.

## 3 Redox regulation of cell fate under ER stress and UPR

**Fig. 3:**
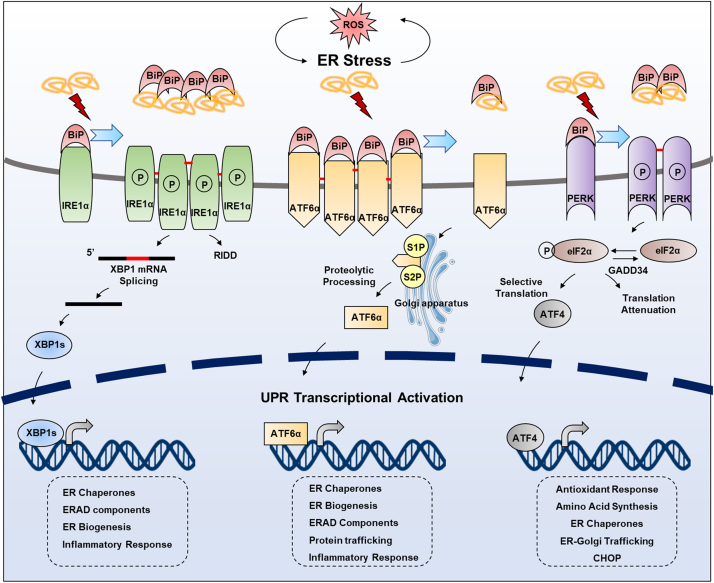
**UPR pathway under ER stress.** Under physiological conditions, BiP binds with and inhibits IRE1α, ATF6α and PERK. When ER stress occurs, the misfolded peptides sequester BiP to release the three UPR transducers. IRE1α undergoes homodimerization and autophosphorylation, subsequently activating the endoribonuclease activity to splice XBP1 mRNA. The encoded XBP1s from spiced mRNA acts as a transcription factor to induce transcription of various target genes. ATF6 oligomer that exists under normal condition is reduced and exported from the ER to the Golgi, where it is proteolysed by Golgi-resident proteases, S1P and S2P. The truncated ATF6 translocates to the nucleus and induces transcription of target genes. PERK activation requires oligerization and autophosphorylation, which obtains the ability to phosphorylate eIF2α. Phosphorylated eIF2α transiently downregulates protein synthesis to decreasing ER protein load, or selectively translates ATF4, which is a transcription factor for a series of UPR target genes.

Perturbation in ER homeostasis (protein folding homeostasis or redox homeostasis) could result in ER stress, a condition under which misfolded and unfolded proteins accumulate in the ER. ER stress is characterized by the induction of UPR. Stressed cells initiate UPR to cope with ER stress, which is well known to relieve protein folding stress by increasing protein folding capacity and decreasing protein folding load [67]. The UPR consists of a complex network of interconnected signaling pathways initiated by the stimulation of three signal transducers located in the ER. These sensors, the major ER-spanning transmembrane proteins, are IRE1α, PERK, and ATF6α, each of which is bound by the ER chaperone BiP under physiological conditions, being locked in monomeric, inactive states [17], [68]. When ER stress occurs, this association will be gradually broken by the enhancement of ER stress and the three sensors are activated (Fig. 3).

Activated IRE1α is in oligomerization and is autophosphorylated by itself due to its kinase activity to facilitate performing the RNase activity, then IRE1α specially splices a 26 nucleotides intron of downstream X-box binding protein 1 (XBP1) mRNA. The spliced mRNA is translated into a specific protein, XBP1s, which is a potent transcription factor and subsequently be translocated into the nucleus to initiate transcription of related genes responsible for regulating ER quality control and ERAD pathway [69], [70], [71]. In addition to XBP1, IRE1α also targets other transcripts through a process called regulated IRE1-dependent decay (RIDD), which participates in maintaining ER homeostasis by reducing ER client protein loading through mRNA degradation [72].

Similar to IRE1α, PERK undergoes oligomerization and autophosphorylation during ER stress [73]. Then, activated PERK leads to phosphorylation of eukaryotic translation initiation factor 2α (eIF2α) on Ser51, which is required for transient downregulation of protein synthesis for abnormal ER recovery and selective expression of Activating Transcription Factor 4 (ATF4) to regulate adaptive genes (such as autophagy-related genes etc.) to relieve ER stress [74]. ATF4 also induces the expression of CAAT/enhancer binding protein (C/EBP) homologous protein (CHOP), which is involved in cell death induction [75], [76]. It is worth mentioning that DNA damage gene 34 (GADD34) compromises PERK signaling by inducing dephosphorylation of eIF2α [77], [78].

Activated ATF6α is transported to Golgi apparatus where it is cleaved by two Golgi-resident proteases, site-1 and site-2 proteases (S1P and S2P) [79]. Cleaved-ATF6α acts as a transcription factor translocating into the nucleus to promote UPR gene expression (including XBP1 and ATF4) involved in IREα and PERK signaling [80], [81], [82].

The released ER sensors activate UPR to restore the ER homeostasis [19], therefore maintaining cell growth and survival. If the adaptive responses failed to restore protein folding homeostasis, UPR signaling continues to persist and eventually morphs into alternate signaling programs called the ‘‘terminal UPR’’ that ultimately promote apoptosis [83], [84]. Growing evidence has shown that UPR plays a pivotal role in cell death or survival determination, which involves several important cellular processes, including antioxidant response, induction of autophagy, apoptosis, or ferroptosis, etc. ROS, which are mainly produced as a byproduct of disulfide bonds formation, might be sensed by stress sensors in the ER lumen and modulate the UPR signaling pathway. Therefore, the integrated action of ER/redox signaling pathway represents an important and intricate feature of cells' capability to cope with their environment to determine the state and fate of cell.

### 3.1 UPR and antioxidant response

The role of UPR in maintaining redox homeostasis is attracting great interest recently [85]. PERK, one of the three UPR transducers, has been reported as a direct upstream molecule for the transcription factor nuclear factor erythroid 2-related factor 2 (NRF2), which is the best-characterized antioxidant transcription factor to counterbalance the harmful effects of ROS in cells [86]. Under ER stress, NRF2 is phosphorylated by the PERK, resulting in NRF2 dissociation from KEAP1 [87]. The liberated NRF2 migrates to the nucleus where it transcripts genes harboring an antioxidant response element in their promoter region. NRF2 can not only induce a panel of genes encoding antioxidant factors, but also repress the transcription of genes encoding pro-oxidant proteins, such as thioredoxin interacting protein (TXNIP) [88]. In addition, previous reports have shown that the heterodimer consisting of NRF2 and ATF4, binds to the stress-response element to induce heme oxidase-1 gene expression [89]. These studies indicate that PERK-NRF2 flux of UPR is a pivotal signaling pathway to restore the redox homeostasis in the ER, unrevealing a UPR-regulated antioxidant mechanism to keep ER redox homeostasis.

### 3.2 Redox regulation between ER stress and autophagy

#### 3.2.1 UPR and autophagy

Autophagy can be activated by ER stress, to eliminate damaged ER (a process termed ER-phagy) and abnormal protein aggregates through the lysosomal pathway [90], [91]. Activation of the autophagy can be mediated by c-Jun NH2-terminal kinase (JNK), which is downstream of IRE1α during ER stress. JNK phosphorylates and activates Bcl-2. The activated Bcl-2 results in the disruption of the Beclin1/Bcl-2 complex, thus releasing Beclin1 to form the vacuolar protein sorting 34 (Vps34)-Beclin1 complex, which drives the nucleation of the isolation membrane [92], [93]. In addition, XBP1, the transcription factor activated by IRE1α RNase domain, is found to trigger autophagy through transcriptional activation of Beclin1 [94].

The activation of PERK inhibits general protein translation by eIF2α phosphorylation, enabling dedicated translation of transcripts harboring an alternate open reading frame, including ATF4, a key UPR transducer [19], [95]. ATF4 transcriptionally regulates Atg12, and ATF4-mediated CHOP activation induces transcription of Atg5. Atg5 and Atg12, together with Atg16L, form Atg5-Atg12-Atg16L complex, which mediates the lipidation of LC3 and elongation process [96]. Moreover, ATF6α, may indirectly regulate autophagy via XBP-1 and CHOP [97].

ER-phagy is a selective autophagy that degrades ER. In recent years, researchers have found that the members of the FAM134 reticulon protein family are ER-resident receptors that bind to autophagy modifiers LC3 and GABARAP, and facilitate ER degradation by autophagy (ER-phagy). Downregulation of FAM134B protein in human cells causes an expansion of the ER, while FAM134B overexpression results in ER fragmentation and lysosomal degradation. Mutant FAM134B proteins are unable to act as ER-phagy receptors. Consistently, disruption of FAM134B in mice causes expansion of the ER, inhibits ER turnover, and sensitizes cells to stress-induced apoptotic cell death. It can be affirmed that selective ER-phagy is indispensable for cell homeostasis and controls ER morphology and turnover under ER stress. However, the crosstalk between ER stress and ER-phagy remains elusive [98], [99], [100].

#### 3.2.2 Redox signaling from UPR to autophagy

**Fig. 4:**
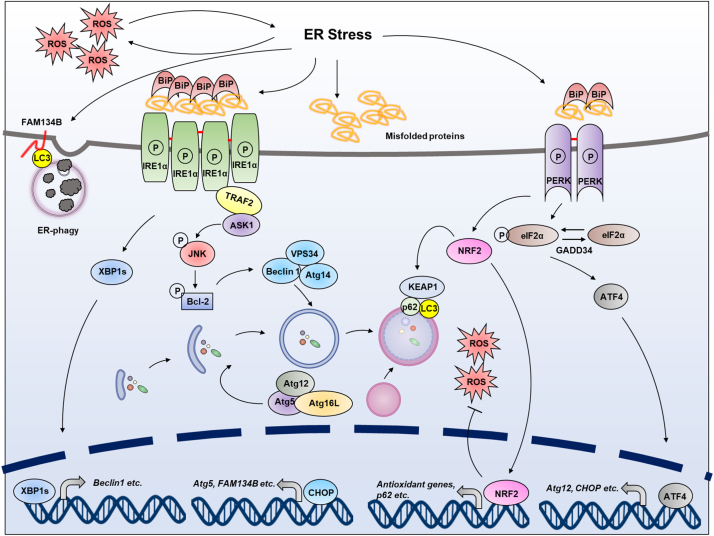
**Redox regulation of autophagy under ER stress.** In certain conditions, the damage of ER and the disturbance of ER homeostasis caused by ER stress or oxidative stress are the main causes of autophagy (including ER-phagy). Autophagy is activated by JNK, which is regulated by IRE1α during ER stress. JNK mediates phosphorylation of Bcl-2, which results in the disruption of the Beclin1/Bcl-2 complex, thus releasing free Beclin1 to form the Vps34-Beclin1 complex. Vps34-Beclin1 complex then drives the nucleation of the isolated membrane to form autophagosome. In addition, XBP1, the transcription factor which is mediated by IRE1α RNase domain, also triggers autophagy through transcriptional activation of Beclin1. PERK signaling cascade can regulate autophagy through ATF4, CHOP and NRF2. ATF4 and CHOP transcriptionally upregulate various autophagy-related genes, such as Atg5, Atg12, and FAM134B (ER-phagy-related gene). NRF2 can be activated by oxidative stress, which induces p62/SQSTM1 binding to KEAP1 and promotes NRF2 release.

Under oxidative stress condition, misfolded proteins induced by ER stress upregulate the autophagy flux, leading to elimination of non-functional and potentially damaging protein aggregates and affected ER. Several groups have reported redox-modification of autophagy components with a general effect on autophagy induction, and altered redox homeostasis of ER, has been involved in the initiation of autophagy. The occurrence of ER stress will lead to activation of NRF2 by PERK signaling and result in modulation of autophagy [101], [102]. In turn, the autophagy-related protein p62/SQSTM1 can bind to KEAP1 at the NRF2 binding site, thus promoting NRF2 release from KEAP1 and enabling NRF2-dependent gene expression. Binding of p62 to KEAP1 is favored upon phosphorylation of p62 in an mTORC1 dependent manner. One dimer of p62 can bind to both KEAP1 and LC3, resulting in its degradation. Moreover, the p62 gene has been shown to be a target of NRF2, creating a positive feedback loop where p62 modulates NRF2 protein levels, which in turn control p62 gene expression (Fig. 4) [103], [104].

### 3.3 Redox regulation between ER stress and apoptosis

#### 3.3.1 UPR and apoptosis

Prolonged ER stress leads to activation of the pro-apoptotic UPR. Ample evidence supports that two UPR kinases, PERK and IRE1α, engage a distinct set of pro-apoptotic outputs [68], [105]. PERK phosphorylates eIF2α, thereby repressing protein translation but promoting translation of ATF4 [19], [95], [106]. ATF4 then promotes the transcription of CHOP, a bZIP transcription factor [75]. CHOP can induce apoptosis by both downregulating the anti-apoptotic protein Bcl-2 [107], and transcriptionally upregulating the proapoptotic proteins BIM and PUMA [108], [109]. Therefore, these pro-apoptotic proteins, induce the release of cytochrome c and initiate the caspase cascade.

Another apoptotic pathway is apoptosis signal-regulating kinase 1 (ASK1), which is activated by the IRE1α-TNF receptor-associated factor 2 (TRAF2)-ASK1 complex. IRE1α is controlled by various regulatory proteins. The activation of IRE1α by UPR signals leads to oligomerization and autophosphorylation, and the formation of an IRE1α-TRAF2 complex through recruitment of the adaptor protein TRAF2. The downstream kinases ASK1 and JNK are subsequently activated to facilitate the activation of ER stress-induced IRE1α-JNK-mediated apoptosis [71], [110], [111].

#### 3.3.2 Redox signaling from UPR to apoptosis

**Fig. 5:**
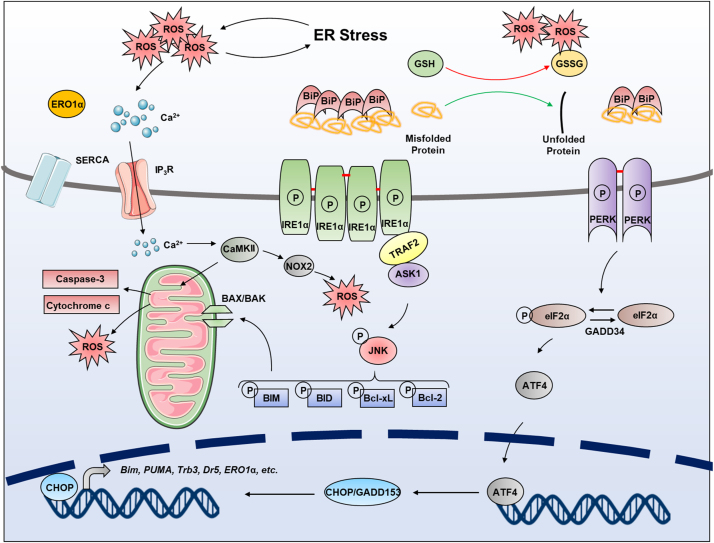
**Redox regulation of apoptosis under ER stress.** PERK can provoke apoptotic signaling by phosphorylating eIF2α, which stimulates the translation of ATF4. Acting as a transcription factor, ATF4 then promotes the transcription of CHOP/GADD153, which downregulates the anti-apoptotic protein Bcl-2 and upregulates the pro-apoptotic proteins BIM and PUMA. CHOP/GADD153 also transcriptionally induces ERO1 to produce ROS. Another apoptotic pathway is the JNK, which is activated by the IRE1α-TRAF2-ASK1 complex. Phosphorylated JNK activates the downstream apoptotic pathways, and induces oxidative stress related to mitochondria. In addition, the accumulation of misfolded proteins induced by ER stress will consume a large amount of GSH to induce oxidative stress. Moreover, calcium channel proteins IP_3_R can be activated by SERCA, leading to calcium outflow which induces apoptosis.

The most direct relationship between ER stress and redox regulation of apoptosis is that many forms of ROS can disturb ER protein folding and induce ER stress. Such as the most common form of ROS (H_2_O_2_), it can stimulate the UPR to cause apoptosis [85]. The loss of GPx7 in mice led to oxidative stress-induced tissue damage and apoptosis, increased tumorigenesis, and impaired longevity [112]. Given the role of disulfide bond formation in the ER as an important source of ROS, protein misfolding in the ER could contribute to oxidative stress. When the microenvironment of ER protein folding is severely disrupted, a futile cycle of disulfide bond formation and reduction could lead to oxidative stress by generating a large amount of ROS and depleting ER GSH levels, which eventually lead to apoptosis [113]. As a major pro-apoptotic factor of the UPR, CHOP also induces oxidative stress in different manners. Excessive activation of ERO1α by CHOP can increase ROS production during ER stress. In addition, ERO1α causes inositol-1,4,5-trisphosphate receptor (IP_3_R)-mediated Ca^2+^ leakage from the ER, which activates Ca^2+^ sensing kinase CaMKII in the cytosol, leading to the activation of pro-apoptotic pathways, including Fas and mitochondrial membrane permeability transition [90], [114], [115]. CaMKII also induces NADPH oxidase subunit NOX2 to cause oxidative stress, which results in PKR-dependent CHOP induction as a positive feed-forward cycle during ER stress (Fig. 5) [116].

### 3.4 Other cellular processes related to UPR and oxidative stress

In addition to the above-mentioned autophagy and apoptosis, which are directly regulated by the three branches of UPR, numerous evidence indicates that various processes involved in cell state and fate decision are related to UPR and oxidative stress.

Ferroptosis, which is a recently recognized form of programmed cell death characterized by the accumulation of lipid peroxidation through ROS generation via the Fenton reaction [117], [118], can be regulated by ER stress and oxidative stress. Ca^2+^ plays a fundamental role in ferroptosis induction through oxidative glutamate toxicity or oxytosis [119], while ER is the most important organelle for Ca^2+^ storage [120], [121], loss of the homeostasis of Ca^2+^ in the ER leads to ER stress [120], [121]. In light of recent studies, ferroptotic agents induce upregulation of PUMA through ER stress-mediated PERK-eIF2α-ATF4-CHOP cascade without inducing apoptosis [22]. Interestingly, oxidative stress can influence key regulators in intracellular Ca^2+^ homeostasis [122]. Oxidative stress increases production of inositol-1,4,5-trisphosphate (IP_3_), which binds and activates IP_3_ receptors 1–3 (IP_3_R1–3) at the ER membrane to trigger Ca^2+^-release from the ER lumen, therefore inducing ER stress [121], [123], [124]. Stromal interaction molecule 1 (STIM1) conveys information of the Ca^2+^ load in the ER lumen to store operated Ca^2+^ channels (SOCs) [125], [126]. ROS can glutathionylate STIM1 at Cys56 to active Ca^2+^ entry [127], [128].

A number of reports have shown that UPR has the potential to induce inflammation via the activation of NF-κB pathway [129]. For example, PERK/eIF2α-induced attenuation of IκBα translation results in dissociation of the NF-κB/IκBα complex, leading to the nuclear translocation of NF-κB and subsequent expression of various genes involved in the inflammatory pathways [130]. Moreover, recent studies have found that UPR-mediated NRF2 activation can effectively inhibit the inflammatory response caused by excessive ROS [129], [131]. In addition to inflammatory regulation, a previous study uncovered that phosphorylated eIF2α protects cells from ROS by inhibiting senescence. Gene knockout of either eIF2α or PERK leads to proliferative defects associated with increased DNA damage, G2/M accumulation and induction of premature senescence [132]. Activation of oncogenes, such as HRAS and c-MYC, has been shown to be able to induce the UPR, leading to premature senescence [133]. In addition, UPR-mediated upregulation of phosphorylated protein kinase B (p-AKT) and downregulation of phosphorylated extracellular signal-regulated kinase (p-ERK) can attenuate senescence in part [134]. Besides regulation of inflammation and senescence, growing evidence has shown that UPR under oxidative stress is associated with various cellular physiological or pathological processes (such as stemness [135], and cell cycle [136], etc.).

## 4 Redox modifications of UPR sensors controlling cell fate

Given the fact that the ER is a cellular component where ROS are generated and scavenged to keep a homeostatic status, as we have discussed, various signaling transducers in the ER, especially those involved in UPR activation, could be redox regulated [85], [137]. The ER stress sensor, including BiP, IRE1α, ATF6α, and PERK, play central roles in cell fate decision [90]. Here, we mainly focus on the redox modifications of these stress sensors in UPR.

### 4.1 Redox regulation of BiP

BiP is a sensor of ER stress. Under ER stress, impaired protein folding in the ER leads to BiP release from ER stress transducers, including PERK and IRE1α [19]. Studies in yeast demonstrate that the interaction between BiP and misfolded proteins is dependent on its ATPase activity [138], which is regulated by the J domain of Sec. 63p [139]. The ATPase domain of BiP contains a conserved cysteine (Cys63) [140], which can be sulfenylated [138] or glutathionylated [60], suggesting a redox-regulated mechanism of BiP release and UPR initiation. The redox regulation of BiP function is delicate. Firstly, the sulfenylation of Cys63 in BiP by ERO1-generated H_2_O_2_ blocks the translocation of nascent proteins into the ER, consequently reduces the folding burden in the ER and production of ROS, thus protecting cells against oxidative stress [139]. Secondly, the sulfenylated BiP loses its ATPase activity [139], releases from PERK and IRE1α, therefore initiating UPR [19], evidenced by the observation that mimics of sulfenylated BiP (BiP-C63D/F/Y/W) induces robust UPR at both permissive (24 °C) and restrictive (37 °C) temperatures compared to wild-type [139].

**Fig. 6:**
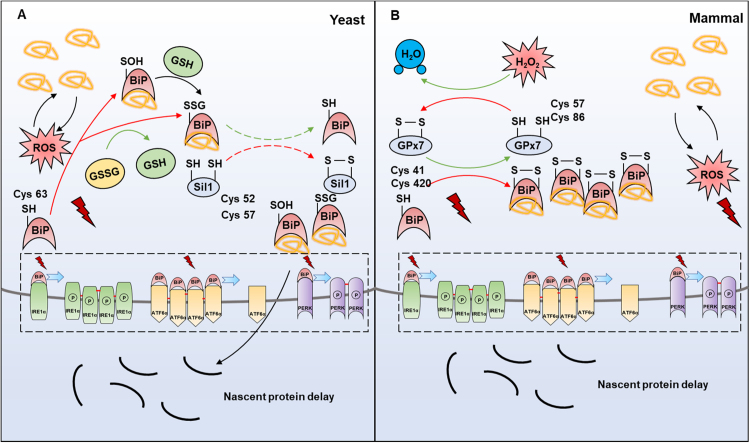
**Redox regulation of BiP to control UPR.** (A) BiP is an ER chaperone that interacts with downstream transducers in the ER. This interaction can be disrupted by the accumulation of misfolded proteins. In yeast, the Cys63 of BiP can be sulfenylated by H_2_O_2_, or glutathionylated by GSSG, which enhances its ability to bind to misfolded proteins. Meanwhile, its ATPase activity is downregulated, resulting in the blockage of nascent proteins’ transportation. Sil1 and BiP exchange disulfide, thereby allowing BiP to restore its ATPase activity. (B) In mammals, the redox modification of BiP is mediated by disulfide-bonded GPx7, which oxidizes BiP to form a disulfide bond between its Cys41 and Cys420 residues.

BiP is also reported to be glutathionylated at Cys63, which shows a similar function as the sulfenylation modification in yeast [142]. As the S-glutathionylation is a reversible oxidative modification, the reductase of BiP has recently been identified to be Sil1. The capacity for Sil1 as a reductase relies on a pair of cysteines (Cys52 and Cys57) within the N-terminus. Sil1 with reduced form of cysteines facilitates glutathione release from the BiP cysteine. Mechanistically, Sil1 reverses BiP glutathionylation through formation of a BiP-Cys63-Sil1-Cys52/Cys57 intermediate, followed by the disulfide bridge formation between Cys52 and Cys57 of Sil1, and the simultaneous reduction of BiP at Cys63 (Fig. 6A) [143]. Notably, glutathione S-transferase Pi is reported to regulate S-glutathionylation of ER-resident proteins, including BiP, therefore promoting cell survival and inhibiting apoptosis under ER stress in mice [60].

In mammals, BiP has two cysteines, Cys41 homologous to Cys63 of yeast BiP, and Cys420. The sulfenylation and glutathionylation of mammalian BiP remain unreported. Nevertheless, redox modification involving Cys41 and Cys420 has been demonstrated, which is mediated by GPx7 [112]. Under oxidative stress, GPx7 forms a disulfide bond between Cys57 and Cys86. Oxidized GPx7 interacts with BiP and forms an inter-molecular disulfide bond between Cys86 of GPx7 and Cys41/Cys420 of BiP. This covalent intermediate subsequently obtains an electron from the solved cysteine of BiP, resulting in the disulfide bond formation between Cys41 and Cys420 of BiP, which enhances its chaperone activity [112]. The anti-oxidative stress function of this modification is similar to that in yeast (Fig. 6B). However, the function of redox regulation of BiP by GPx7 in UPR induction remains uninvestigated.

### 4.2 Redox regulation of IRE1α

**Fig. 7:**
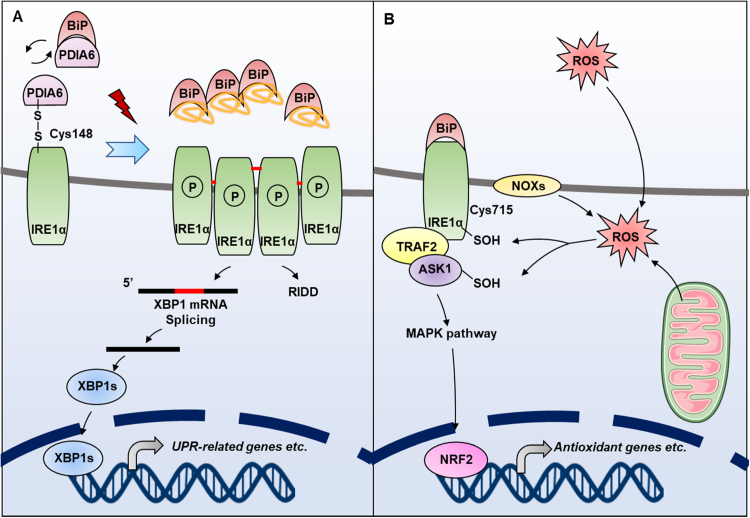
**Redox regulation of IRE1α to control UPR.** (A) Under physiological condition, the Cyst148 of IRE1α forms an intermolecular disulfide bond with PDIA6 and is therefore kept in an inactive state. PDIA6 can also form a complex with BiP by non-covalent bonding to catalyze the oxidative protein folding. Under ER stress, IRE1α forms tetramers through disulfide bonds and is auto-phosphorylated, activating downstream signals. (B) IRE1α is able to sense the level of ROS in the ER or cytoplasm. Under oxidative stress, Cys715 that locates in the cytoplasmic region of IRE1α is sulfenylated by ROS (generated from NOXs, ER as well as mitochondria), thus activating the MAPK pathway to initiate NRF2-mediated antioxidant system, meanwhile inhibiting the UPR-initiating function of IRE1α.

ROS are involved in the UPR-mediated autophagy or apoptosis under therapeutic treatment [144], [145]. The activation of IRE1α is dependent on ROS, as the scavenging of ROS by NAC restores the activation of IRE1α and UPR [145], implying that IRE1α may be redox-regulated under ER stress. When UPR is activated, IRE1α forms oligomer and trans-autophosphorylates itself to drive transcription of UPR target genes, which relieves ER stress, then IRE1α oligomers dissociate consequently [67]. Two intermolecular disulfide bonds were identified as Cys148-Cys148 and Cys332-Cys332, respectively, however, the disulfide bond formation is not required for UPR activation in yeast [146]. Nevertheless, it has been shown that the IRE1α oligomer is a disulfide species that requires Cys148 in the luminal domain, the mutation of which impedes proliferation of MEF [147], implying that IRE1α is oxidized to form or stabilize the oligomer in mammals. More importantly, the function of IRE1α is also regulated by the formation of an inter-molecular disulfide bond between Cys148 of IRE1α and a cysteine of PDIA6 (Fig. 7A) [85], [147]. This disulfide-dependent interaction accelerates dephosphorylation and inactivation of IRE1α, therefore limiting the duration of IRE1α activation, preventing the hyper-activation of UPR and apoptosis [147].

The UPR-initiating function of IRE1α can be interfered by sulfenylation at a highly conserved cysteine (Cys715 in human) [148]. As the cysteine locates in the IRE1α kinase domain, its sulfenylation impaires the kinase activity and inhibits the IRE1α-mediated UPR, but initiates the p38/NRF2 antioxidant response instead, thereby increasing stress resistance and survival. The Cys715 can be sulfenylated by cytoplasmic, ER-contacted mitochondrial, and ER ROS. The cytoplasmic ROS is produced by IRE1α-interacting NOX (BLI3 in *C. elegans*) that is at or near the ER, and ER-contacted mitochondrial ROS is generated from the electron transport chain, while ER ROS comes from ERO1 and/or QSOX (Fig. 7B) [148].

### 4.3 Redox regulation of ATF6α

ATF6α, an ER transmembrane protein, is transported to the Golgi and is cleaved by S1P and S2P to release a 50 KD cytosolic portion, which moves to the nucleus and acts as a transcription factor to transcript UPR genes under ER stress [149], [150], [151]. Under unstressed conditions, ATF6α is disulfide-bonded in the luminal domain by forming inter- and intra-molecular disulfide-bridges, which is mainly dependent on Cys467 and Cys618 [152]. In response to ER stress, disulfide reduction and disassembly of AFT6α are required to mediate its transportation to the Golgi but not sufficient for its activation. The PDI family member PDIA5 is implicated in the reduction of ATF6α disulfides, as silencing of PDIA5 limits disulfide-bonded oligomer dissociation of ATF6α under stress conditions. Although direct electron exchange between ATF6α and PDIA5 has not been demonstrated, it seems likely that PDIA5 knockdown abolishes direct reduction of ATF6α disulfides by PDIA5 [20]. These observations demonstrate that, in contrast to IRE1α, ATF6α undergoes disulfide reduction for activation during ER stress.

### 4.4 Redox regulation of PERK

PERK oligomerizes and autophosphorylates itself under ER stress conditions [19], [153]. Growing evidence indicates that PERK is activated during oxidative stress, meanwhile PERK plays a dual role in cell fate decision. For example, following glucose deprivation-induced ER stress, PERK is activated and then phosphorylates NRF2, which contributes to redox homeostasis and cell survival via antioxidant response [87]. PERK is also critical to convey apoptosis by sustaining the levels of CHOP under ROS-mediated ER stress [154]. Evidence has shown that under glucose deprivation, NOX4-derived ROS can activate PERK by inhibiting the ER-specific PHD4, therefore promoting cardiomyocyte autophagy and survival [7]. These studies imply that PERK may be redox regulated.

Although solid evidence for redox modification of PERK remains insufficient, there are studies supporting the oxidative modification at cysteine(s) of PERK. For example, PDIA6, which facilitates the inactivation of IRE1α through covalent binding via a disulfide bond, is able to functionally associates with PERK, as loss of PDIA6 leads to increased and prolonged phosphorylation of eIF2α [147]. Two other PDI family members, ERp57 (PDIA3) and PDI (PDIA1), are involved in the redox regulation of PERK [155]. ERp57 and PDI play opposing roles in the regulation of PERK function. Depletion of ERp57 results in an intra-molecular disulfide bond formation of PDI, consequently activates PERK, whereas loss of PDI function reduced PERK activation [155]. Considering the ability of PDIs to catalyze disulfide bond formation, it is intriguing to investigate whether PERK is oxidized to form a disulfide bond during its activation.

## 5 Concluding remarks

There is an interesting question on how cells determinate its fate in response to ER stress. ROS are generated in the ER through oxidative protein folding process [4], [5]. The disturbance of protein folding homeostasis or redox homeostasis in the ER will cause ER stress, then an ER-specific process termed UPR is induced to alleviate the stress [156], [157]. When the stress is successfully alleviated, cells will survive because of the UPR-activated pro-survival signaling. However, UPR can also lead to cell death due to the persistence of the stress. Great advances have been made to reveal the roles and mechanisms of ROS or oxidative stress in regulating UPR. As we discussed in this review, the regulation of UPR largely involves the redox modifications of ER stress sensors, which finely tunes UPR to activate pro-survival or cell death signaling. This raises another interesting question: what is the difference between UPR that exert pro-survival or pro-apoptotic process? It is commonly thought that severe or persistent ER stress may provoke apoptotic cell death [68], [157], [158]. However, whether the cell fate is determined at a switching point from survival to death, or as a compromising consequence of co-existing cellular processes (including but not limited in autophagy and apoptosis), remains to be investigated.
